# Case of Strumous Suppuration of the Synovial Capsule of the Hip-Joint, Treated with Iodine

**Published:** 1829-12

**Authors:** William John Thomas


### DISEASE OF THE HIP-JOINT.

*Case of Strumous Suppuration of the Synovial Capsule of the Hip-joint, treated with Iodine.* By William John Thomas, M.R.C.S.

Master A. B., aged seven years, was brought to me on the 26th April, 1829, for advice respecting a tumor which had arisen in the groin, a week or two previously, and which had considerably enlarged since its first appearance. He was of a strongly-marked strumous diathesis: the skin was smooth and delicate, the complexion fair; the hair of a light colour, soft, and silky; the irides were blue, the upper lip was thick and projecting, and the cheeks of a peculiar rosy hue.

Upon examining the tumor, an obscure fluctuation was perceptible: suspecting that it might arise from a psoas abscess, I carefully examined the lumbar vertebræ, but could detect no preternatural sensibility. The hip-joint, however, was somewhat tender upon pressure, especially around the trochanter major, and there was some appearance of swelling over the glutei muscles. Aperient medicines were prescribed, and a poultice ordered to the tumor.

On the 18th May I punctured the abscess. He recovered a temporary use of the limb, and went into the country for the benefit of his health.

About the commencement of July, I was again desired to visit him. He had returned from his excursion in a considerably worse condition than he had departed. The thigh was now permanently bent at nearly a right angle with the body. He complained of exquisite pain upon the slightest movement of the leg; and, upon examination, another inguinal abscess was detected, somewhat lower than the site of the original tumor. This was allowed to burst under the use of fomentations and poultices; the discharge exhibiting the peculiar curdy matter of strumous suppuration. The parts around the great trochanter of the femur were considerably inflamed, and motion in the acetabulum aggravated the permanent pain in the joint itself. Leeches were therefore applied every third day until the local inflammation was suppressed. As the active inflammation subsided, I was desirous of trying the effects of counter-irritation, and proposed blisters. Their application, however, was objected to by the parents, who were fearful of inflicting pain upon the child. Issues were also opposed upon the same principle.

Under these circumstances I prescribed six drops of the tincture of iodine ter. die. During the administration of this medicine, a third abscess formed, of great extent, beneath the fascia lata. This membrane consequently became greatly distended by the contained matter. By the diligent use of fomentations, &c. the integuments ulcerated below the insertion of the tensor vaginæ femoris, and a large quantity of flaky pus was discharged. At this period the posture of the patient was sedentary, with the trunk inclined forwards, and the femur forming an acute angle with the body. When he stood upon the sound leg, and the heel of the diseased one was gently elevated, he complained of severe pain in the hip-joint, which became intolerable on the slightest rotation of the limb. He was occasionally distressed with pain in the knee, but it never affected him very severely during the progress of the complaint. Periodical paroxysms of hectic fever took place after the bursting of the third abscess, and the constitutional irritation was severe.

The dose of the tincture of iodine varied from five to seven drops twice or thrice a day. I was determined to saturate the system fully with this medicine, and correct, if possible, the vitiated composition of the blood. He pursued this system regularly for ten or twelve weeks; aperients being occasionally prescribed. The sanious and sero-purulent discharge from the abscess became more laudable in appearance, and uniform in consistence. The patient gradually assumed a more healthy aspect as the constitutional sympathies subsided. A liberal diet was then ordered, with animal food and porter.

Under this treatment he rapidly improved, and was soon able to move about on crutches. At present (about eight months from the discovery of the disease) he daily walks to and from school, nearly a mile, morning and evening. He walks unsupported, and, when inclined to do so, he can run fast, and enter into the active sports of children, as usual.

In several other scrofulous cases, I have administered iodine with occasional success, in medium doses, duly persisted in. By thus saturating the system with the medicine, I have seen inflammatory affections of the periosteum of the metacarpal bones, and also incipient necrosis of other osseous textures gradually subside, and a salutary action of the capillary vessels supersede their previously morbid condition.

I shall conclude this communication with a few brief remarks upon strumous diseases in general.

In the first place, there appears to be an hereditary predisposition to this disease in the individuals affected by it. This predisposition may be defined to consist in a peculiar organic construction and conformation of the capillaries of the sero-sanguiferous system, producing an inertia in the functions of those capillary organs, whereby they become incapable of offering a salutary opposition to a morbidly irritating and exciting cause.

Secondly, this latent predisposition runs into diseased action, being morbidly developed by the vitiated contents of the sero-sanguiferous system acting upon their containing organs; thus producing a proximate cause for the manifestation of the morbid effects, and the several consecutive phenomena of scrofulous inflammation.

Lastly, the remote cause of the developement of this strumous predisposition may be referred to the vitiated contents of the prima via, and consequently of the disordered functions of those organs employed in the digestion of the food; therefore, the preliminary operations of the sanguification of the chyle being materially impeded, the blood prepared for the support of the system will be supplied of diminished quantity and diseased quality.

From these propositions it will be obviously deduced, that, to remove the exciting causes; to amend the diseased quality of the blood; to restore general constitutional vigor, and to obviate all preternatural local determinations, are the principal indications to be fulfilled in the treatment of scrofulous diseases.

*Liverpool; November 5th,* 1829.

